# The Prognostic Value of Pretherapy Peripheral Blood Inflammatory Indices in Myelodysplastic Syndromes

**DOI:** 10.3389/fonc.2022.877981

**Published:** 2022-04-26

**Authors:** Cong Shi, Shengping Gong, Tingting Niu, Tongyu Li, An Wu, Xiaojiao Zheng, Shujun Yang, Guifang Ouyang, Qitian Mu

**Affiliations:** ^1^ Stem Cell Transplantation Laboratory, Ningbo First Hospital, Ningbo, China; ^2^ Cancer Radiotherapy and Chemotherapy Center, Ningbo First Hospital, Ningbo, China; ^3^ Department of Hematology, Ningbo First Hospital, Ningbo, China; ^4^ Department of Obstetrics and Gynaecology, Ningbo First Hospital, Ningbo, China

**Keywords:** myelodysplastic syndrome, IPSS-R, prognosis, PLR, NLR, CRP

## Abstract

**Background:**

Inflammation appears to have a critical role in carcinogenesis tumor growth according to emerging research. The platelet-to-lymphocyte ratio (PLR), neutrophil-to-lymphocyte ratio (NLR), and plasma C-reactive protein (CRP) are considered to reflect the systemic inflammatory response and clinical prognosis. The prognostic value of inflammatory indices in myelodysplastic syndrome (MDS) patients remains unclear.

**Methods:**

A total of 213 MDS patients were enrolled for the study. Univariate and multivariate analyses were performed to determine the prognostic significance of various indicators, including PLR, NLR, and CRP.

**Results:**

MDS patients with higher PLR, NLR, and CRP levels had significantly shorter overall survival (OS). Based on univariate analysis, age (≥60 years), gender (men), lower hemoglobin level (<10 g/dl), higher bone marrow blast percentage (>5%), poorer karyotype, and higher Revised International Prognostic Scoring System (IPSS-R) score were significantly associated with shorter OS. Patients with higher CRP levels had shorter leukemia-free survival (LFS, *P* = 0.041). However, higher PLR and NLR had no significant influence on LFS (*P* > 0.05). Multivariate Cox proportional hazards regression analysis indicated that high PLR and CRP were also independent adverse prognostic factors for OS in MDS.

**Conclusions:**

Elevated PLR and CRP predict poor prognosis independent of the IPSS-R and provide a novel evaluation factor for MDS patients.

## Background

Myelodysplastic syndromes (MDSs) are composed of a heterogeneous group of hematopoietic stem cell malignancies characterized by ineffective hematopoiesis manifested by morphologic dysplasia in hematopoietic cells and peripheral cytopenia and have a substantial risk of progression to acute myeloid leukemia (AML) (1). Thus, a reliable prediction model would be a crucial cornerstone for guiding the clinical management of MDS patients. In the past decades, different scoring systems have been introduced to risk-stratify patients with MDS, including the International Prognostic Scoring System (IPSS) in 1997, the World Health Organization (WHO) Classification-Based Prognostic Scoring System (WPSS) in 2007, the MD Anderson Risk Model Score (MDAS) in 2008, and the Revised IPSS (IPSS-R) in 2012 (2–5). The most commonly used grading system for predicting outcomes and tailoring therapeutic approaches is the IPSS-R. Novel biological indicators are being investigated to establish the best treatment plan and assess the prognosis of MDS patients due to the ambiguity regarding their prognosis. Hematologic, morphologic, and cytogenetic characteristics have all been incorporated into clinical grading systems thus far. Low absolute lymphocyte count (ALC), low absolute monocyte count (AMC), and elevated mature monocytes in bone marrow were associated with poor prognosis in MDS (6–8). IPSS-R has no bearing on these variables. IPSS-R does, after all, have its limits.

The tumor microenvironment interacts tightly with tumor cells and is crucial to tumorigenesis and tumor development. MDS is a heterogeneous set of cancers resulting from distorted hematopoietic stem cell function, inflammatory and innate immunological dysregulation, and numerous genetic events, according to some investigations (9–11). About 10%–20% of MDS patients might suffer from systemic inflammation (12). A history of some illnesses, according to the report by Sigurdur et al. (13), raises the incidence of AML and MDS. Platelet-to-lymphocyte ratio (PLR), neutrophil-to-lymphocyte ratio (NLR), and C-reactive protein (CRP), which are inflammatory biomarkers extracted from the peripheral blood, have shown predictive significance in patients with gastric cancer, pancreatic cancer, and renal cell carcinoma (14–16). According to recent studies, inflammation and the immunological microenvironment have a role in the etiology of MDS (17). The inflammatory response is closely linked to cancer pathophysiology and can be reflected by inflammation indicators, including lymphocyte count and platelet count that have been investigated in a variety of malignancies. The application of PLR, NLR, and CRP as prognostic markers for MDS has just a few publications in the literature.

Therefore, our study aimed to evaluate the potential prognostic values of different inflammatory indices including PLR, NLR, and CRP in MDS patients.

## Materials and Methods

### Patients and Clinical Variables

Clinical and follow-up data of 231 patients who were newly diagnosed with MDS in Ningbo First Hospital from 2009 to 2019 were collected. Three patients with malignant diseases and 15 patients with inflammation and autoimmune diseases at the time of diagnosis were excluded. The final sample of 213 patients was included in the study. The candidate prognostic factors of interest in this study were PLR, NLR, and CRP, as well as platelet (PLT), neutrophil (NE), hemoglobin (HB), the percentage of blasts in the bone marrow, and the chromosome subtype-based score, which are known prognostic factors based on the IPSS-R. In addition, age and gender were also investigated in this retrospective study. The diagnosis of MDS and leukemic transformation were based on the 2016 WHO classification (18). Risk stratifications of MDS were made according to IPSS-R.

All indicators involved in the estimation of inflammation-based prognostic scores were derived before treatment. Complete blood count was measured using a fully automated XN-1000 and XN-9000 hematology analyzer system (Sysmex, Japan). CRP was measured using Aristo (Aristo, China). Bone marrow myeloid cells were exposed to Wright–Giemsa stain and evaluated by two laboratory technicians. Cytogenetic analyses on bone marrow cells were conducted for 180 patients. Chromosomes of bone marrow cells were tested using the R-banding method after a 24-h culture. When available, at least 20 metaphases were determined according to the 2016 International System for Human Cytogenetic Nomenclature (ISCN2016) (19). According to IPSS-R, the karyotypes were classified into five categories: very good, good, intermediate, poor, and very poor.

The molecular assessment was conducted as part of a standard clinical examination. Between 2009 and 2018, mutational analysis for 14 common genes of MDS that included *NRAS*, *DNMT3A*, *SF3B1*, *IDH1*, *IDH2*, *TET2*, *EZH2*, *JAK2*, *CBL*, *ETV6*, *TP53*, *SRSF2*, *ASXL1*, and *RUNX1* was conducted using next-generation sequencing. Since the beginning of 2018, 34 common genetic mutations were analyzed using the same method that are *NRAS*, *DNMT3A*, *SF3B1*, *IDH1*, *IDH2*, *TET2*, *EZH2*, *JAK2*, *CBL*, *ETV6*, *TP53*, *SRSF2*, *ASXL1*, *RUNX1*, *KIT*, *KRAS*, *NF1*, *NPM1*, *PHF6*, *PIGA*, *PTPN11*, *SETBP1*, *STAG2*, *U2AF1*, *WT1*, *ZRSR2*, *BCOR*, *BCORL1*, *CALR*, *CEBPA*, *CSF3R*, *MPL*, *ETNK1*, and *FLT3*. Variants having a variant allele frequency of less than 1% were excluded from the analysis. To amplify and assemble the sample collection, multiplex PCR was employed. High-throughput sequencing was performed on the Ion Proton platform. And bioinformatics analysis was done using the PolyPhen, HG19, 1000 genomes, COSMIC, ClinVar, and dbSNP databases. Kindstar Global Medical Laboratory (Wuhan, China) completed the gene mutation detection process.

### Treatment for Patients

Almost all of the patients received symptomatic and supportive care. Seventy-two patients acquired further treatment, of whom 59 individuals (27.7%) were treated with intensive chemotherapy, 17 patients (8.0%) with hemopoietic stem cell transplantation (HSCT), and 30 patients (14.1%) with hypomethylating drugs. Some patients received more than one treatment.

This study was approved by the ethics committee of Ningbo First Hospital (approval number RS286) and conformed to the principles of the Helsinki Declaration throughout the study.

### Statistical Analysis

Statistical analyses were performed by the SPSS software (version 26.0). Differences in the distribution of continuous variables between categories were analyzed by Mann–Whitney *U* test and categorical variables by chi-square test. OS was calculated from the date of initial diagnosis of MDS to the date of death, last follow-up, or receiving allo-HSCT. Leukemia-free survival (LFS) was determined from the date of diagnosis to the date of leukemia transformation. OS and LFS were analyzed using the Kaplan–Meier method and were compared using the log-rank test. Cox regression was used for univariate and multivariate analyses. The hazard ratio and 95% confidence hazards model were used in the analysis. The cutoff point of PLR and NLR was calculated using the *X*-Tile software (20). The optimal cutoff value for differences in survival was selected (the lowest *P*-value under the log-rank test). The reference range of CRP was 0–5 mg/L in our laboratory. The optimal cutoff values were 22.4 for PLR, 3.75 for NLR, and 5 mg/L CRP. *P*-value <0.05 was considered statistically significant.

## Results

### Patient Characteristics

Baseline characteristics of the study population are listed in **Table 1**. The data of the 213 MDS patients, including 89 women and 124 men, were collected over a 10-year period with a median age of 62 years (range 16–90 years). Among these MDS patients, the median OS was 26 months (range 0–125 months, 95% CI 15.1–36.9 months), and 26 patients (12.2%) eventually converted into AML. Albeit receiving active treatments,113 patients died in the end, with a mortality of 53%. Based on the 2016 WHO classification, all patients were classified as MDS as follows: 18 (8.5%) with MDS with single-lineage dysplasia (MDS-SLD), 59 (27.7%) with MDS with multilineage dysplasia (MDS-MLD), 14 (6.6%) with MDS with ring sideroblasts (MDS-RS), 57 (26.8%) with MDS with excess blasts (MDS-EB)1, 46 (21.6%) with MDS-EB2, 6 (2.8%) with MDS-del(5q) including del(5q) alone or with 1 additional abnormality except -7 or del(7q), and 13 (6.1%) with unclassifiable MDS (MDS-U). Besides, IPSS-R classified 180 patients into the following risk groups: 9 (5%) in very low risk, 32 (17.8%) in low risk, 62 (34.4%) in intermediate risk, 41 (22.8%) in high risk, and 36 (20%) in very high risk. The median IPSS-R score was 4.5 (1.0–10.0). Detailed information was also provided in **Table 1**.

**Table 1:** Baseline patient characteristics according to total patients, PLR, NLR, and CRP levels.

Characteristics	Total patients	Patients grouped by PLR level (n = 213)		*P*-value	Patients grouped by NLR level (n = 213)		*P*-value	Patients grouped by CRP level (n = 213)		*P*-value
		PLR ≤22.14 (n = 51)	PLR >22.14 (n = 162)		NLR ≤3.75 (n = 195)	NLR >3.75 (n = 18)		CRP ≤5 mg/L (n = 117)	CRP >5 mg/L (n = 96)	
Men/Women, n	124/89	28/23	96/66		111/84	13/5		62/55	62/34	
Age, years median (range)	62 (16–90)	54 (16–81)	64 (16–90)	<0.0001	62 (16–90)	65 (46–83)	0.349	61 (16–90)	63 (24–85)	0.153
BM blast, % median (range)	4.5 (0–19.5)	2.5 (0–19.5)	5.5 (0–19.5)	0.007	4.5 (0–19.5)	5 (0–13)	0.936	3 (0–19)	6.3 (0–19.5)	<0.0001
**Peripheral Blood**										
NE, ×10^9^/L median (range)	1.2 (0.1–7.4)	1.2 (1.1–6.9)	1.1 (1.1–7.4)	0.461	1.1 (1.1–6.9)	3.7 (1.7–7.4)	<0.0001	1.2 (0.1–6.9)	1.2 (0.1–7.4)	0.682
HB, g/L median (range)	7.5 (2.2–14.2)	6.8 (2.3–13.6)	7.8 (2.2–14.2)	0.024	7.5 (2.2–14.2)	6.9 (3.1–12)	0.397	8 (3.5–14.2)	7.1 (2.2–13.6)	0.032
PLT, ×10^9^/L median (range)	51 (2–332)	17 (2–94)	63.5 (12–332)	<0.0001	51 (2–332)	56 (10–306)	0.834	59 (6–322)	45 (2–332)	0.022
ALC, ×10^9^/L median (range)	1.0 (0.2–5.4)	1.3 (0.5–5.4)	1.0 (0.2–2.8)	<0.0001	1.1 (0.2–5.4)	0.7 (0.2–1.2)	<0.0001	1.1 (0.2–2.8)	1 (0.2–5.4)	0.083
**2016 WHO classification**				0.018			0.607			0.004
MDS-SLD, % (n/n)	8.5% (18/213)	11.8% (6/51)	7.4% (12/162)		8.7% (17/195)	5.6% (1/18)		9.4% (11/117)	7.3% (7/96)	
MDS-MLD, % (n/n)	27.7% (59/213)	33.3% (17/51)	25.9% (42/162)		26.7% (52/195)	38.9% (7/18)		33.3% (39/117)	20.8% (20/96)	
MDS-RS-SLD, % (n/n)	2.3% (5/213)	2% (1/51)	2.5% (4/162)		2.6% (5/195)	0% (0/18)		2.6% (3/117)	2.1% (2/96)	
MDS-RS-MLD, % (n/n)	4.2% (9/213)	2% (1/51)	4.9% (8/162)		4.6% (9/195)	0% (0/18)		6.8% (8/117)	1% (1/96)	
MDS-5q-, % (n/n)	2.8% (6/213)	0% (0/51)	3.7% (6/162)		3.1% (6/195)	0% (0/18)		5.1% (6/117)	0% (0/96)	
MDS-EB1, % (n/n)	26.8% (57/213)	19.6% (10/51)	29% (47/162)		25.6% (50/195)	38.9% (7/18)		20.5% (24/117)	34.3% (33/96)	
MDS-EB2, % (n/n)	21.6% (46/213)	15.7% (8/51)	23.5% (38/162)		22.1% (43/195)	16.7% (3/18)		15.4% (18/117)	29.2% (28/96)	
MDS-U, % (n/n)	6.1% (13/213)	15.7% (8/51)	3.1% (5/162)		6.7% (13/195)	0% (0/18)		6.8% (8/117)	5.2% (5/96)	
**IPSS-R cytogenetic risk group**				0.626			0.575			0.296
Very good, % (n/n)	1.7% (3/180)	0% (0/38)	2.1% (3/142)		1.8% (3/166)	0% (0/14)		1.9% (2/107)	1.4% (1/73)	
Good, % (n/n)	62.2% (112/180)	71.1% (27/38)	59.9% (85/142)		60.8% (101/166)	78.6% (11/14)		64.5% (69/107)	58.9% (43/73)	
Intermediate, % (n/n)	21.1% (38/180)	15.8% (6/38)	22.5% (32/142)		21.7% (36/166)	14.3% (2/14)		23.4% (25/107)	17.8% (13/73)	
Poor, % (n/n)	5% (9/180)	2.6% (1/38)	5.6% (8/142)		4.8% (8/166)	7.1% (1/14)		3.7% (4/107)	6.8% (5/73)	
Very poor, % (n/n)	10% (18/180)	10.5% (4/38)	9.9% (14/142)		10.8% (18/166)	0% (0/14)		6.5% (7/107)	15.1% (11/73)	
**IPSS-R risk category**				0.248			0.477			0.028
Very low, % (n/n)	5% (9/180)	0% (0/38)	6.3% (9/142)		4.2% (7/166)	14.3% (2/14)		6.5% (7/107)	2.7% (2/73)	
Low, % (n/n)	17.8% (32/180)	15.8% (6/38)	18.3% (26/142)		17.5% (29/166)	21.4% (3/14)		22.4% (24/107)	11% (8/73)	
Intermediate, % (n/n)	34.4% (62/180)	50% (19/38)	30.2% (43/142)		35.5% (59/166)	21.4% (3/14)		35.5% (38/107)	32.9% (24/73)	
High, % (n/n)	22.8% (41/180)	13.2% (5/38)	25.4% (36/142)		22.9% (38/166)	21.4% (3/14)		22.4% (24/107)	23.3% (17/73)	
Very high, % (n/n)	20% (36/180)	21.1% (8/38)	26.8% (28/142)		19.9% (33/166)	21.4% (3/14)		13.1% (14/107)	30.1% (22/73)	
**IPSS-R score, median (range)**	4.5 (1–10)	4.0 (2–10)	4.5 (1–10)	0.660	4.5 (1–10)	4.3 (2–7)	0.525	4 (1–10)	5.5 (2–10)	0.002
**Gene mutation, % (n/n)**	61.7% (37/60)	33.3% (3/9)	63% (34/51)	0.074	62.5% (35/56)	50% (2/4)	0.634	55% (22/40)	75% (15/20)	0.133
**Leukemia transformation, % (n/n)**	12.2% (26/213)	9.8% (5/51)	13% (21/162)	0.548	13.3% (26/195)	0% (0/18)	0.138	8.5% (10/117)	16.7% (16/96)	0.072
**Complex karyotype, % (n/n)**	18.9% (34/180)	15.8% (6/38)	19.7% (28/142)	0.583	19.9% (33/166)	7.1% (1/14)	0.474	15% (16/107)	24.7% (18/73)	0.102

BM, bone marrow; NE, neutrophil; HB, hemoglobin; PLT, platelet; ALC, absolute lymphocyte count; PLR, platelet-to-lymphocyte ratio; NLR, neutrophil-to-lymphocyte ratio; CRP, C-reactive protein; MDS-SLD, MDS with single-lineage dysplasia; MDS-MLD, MDS with multilineage dysplasia; MDS-RS-SLD, MDS with ring sideroblasts and single-lineage dysplasia; MDS-RS-MLD, MDS with ring sideroblasts and multilineage dysplasia; MDS-EB1, MDS with excess blasts 1; MDS-EB2, MDS with excess blasts 2; MDS-U, unclassifiable MDS; IPSS-R, Revised International Prognostic Scoring System.

### Correlation of Platelet-to-Lymphocyte Ratio, Neutrophil-to-Lymphocyte Ratio, and C-Reactive Protein With Clinical and Laboratory Factors

In our cohort, MDS patients were divided into two groups to analyze the correlation between PLR, NLR, and CRP levels and clinical and laboratory characteristics. It showed that patients with higher PLR had significantly more counts of bone marrow (BM) blast (*P* = 0.007) and PLT (*P* < 0.0001) with concomitantly lower levels of HB (*P* = 0.024) and ALC (*P* < 0.0001) compared with those with lower PLR. The higher NLR group had significantly higher NE (*P* < 0.0001) and lower ALC (*P* < 0.0001). The higher CRP group had significantly more BM blast counts (*P* < 0.0001) and less HB (*P* = 0.032) and PLT (*P* = 0.022) counts. In addition, CRP was closely associated with the IPSS-R risk category, as patients with higher IPSS-R scores exhibited higher CRP levels. There were no significant differences in other factors. The association of PLR, NLR, and CRP with the clinical and laboratory characteristics was also listed in **Table 1**.

### Correlation of Platelet-to-Lymphocyte Ratio, Neutrophil-to-Lymphocyte Ratio, and C-Reactive Protein With Gene Mutations

**Figure 1:**
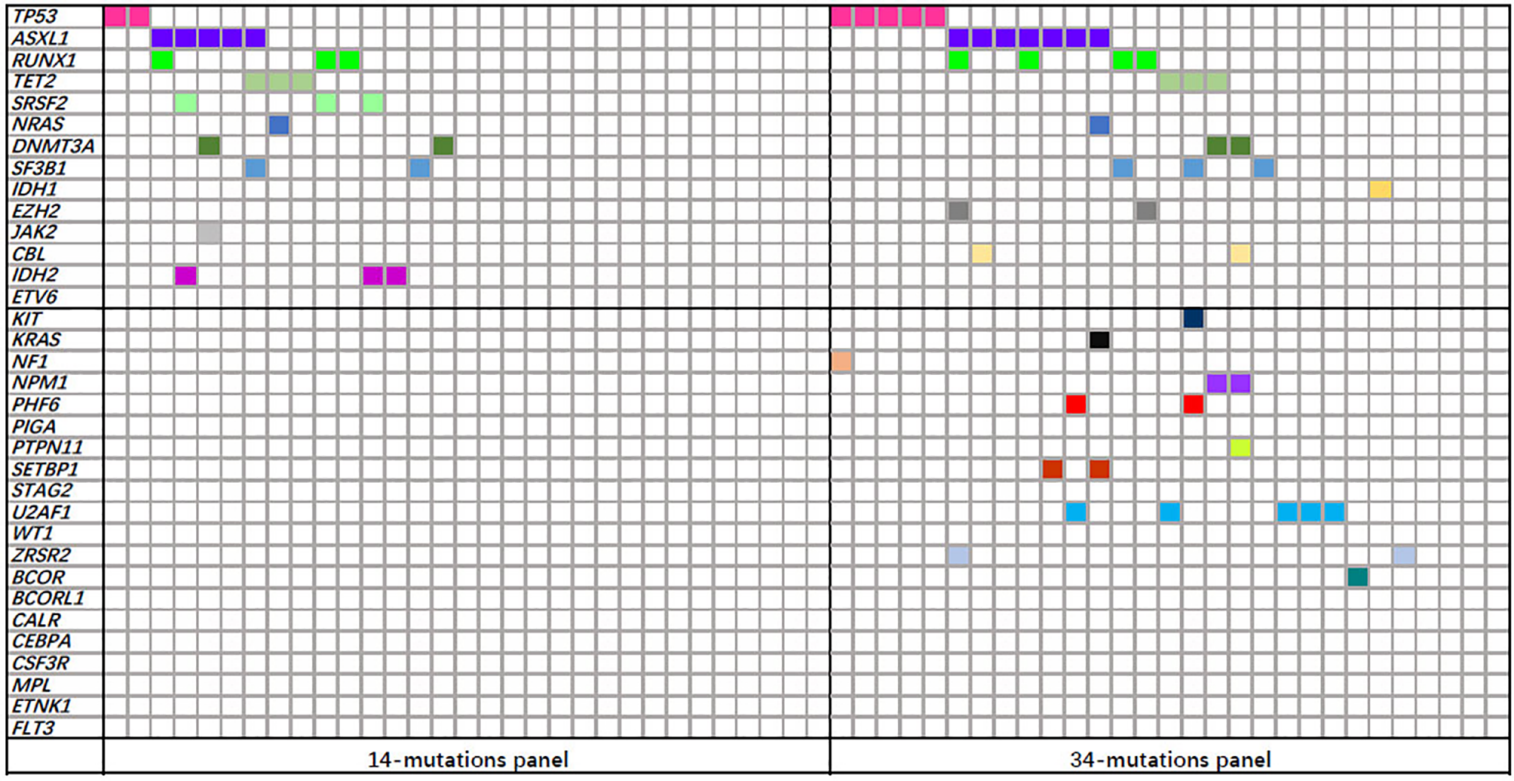
Mutation spectrum of 14 or 34 common genes in 60 myelodysplastic syndrome (MDS) patients. Each column represents an individual patient sample, and each colored cell represents a mutation of the gene.

A total of 60 MDS patients were exposed to gene sequencing to increase the accuracy of risk categorization. Among these patients, 31 were evaluated using a 14-mutation panel, and 15 (48.4%) were found to harbor more than one gene mutation. The other 29 individuals were exposed to the detection of a panel of 34 mutations, and 25 (86.2%) were identified to carry varied gene mutations. In all, 40 individuals had genetic alterations, with 20 having only one mutation, 11 having two mutations, and 9 having three or more mutations. **Figure 1** showed the mutation spectrum of 14 or 34 common genes in 60 MDS patients. However, the levels of PLR, NLR, and CRP had no significant correlations with these mutated genes (data not shown).

### The Association Between Inflammatory Biomarkers and Prognosis

**Figure 2:**
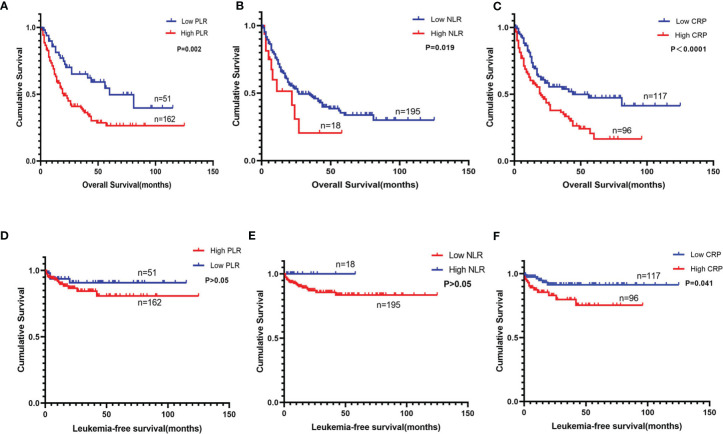
Overall survival and leukemia-free survival of myelodysplastic syndrome (MDS) patients according to the stratified analysis of platelet-to-lymphocyte ratio (PLR), neutrophil-to-lymphocyte ratio (NLR), and C-reactive protein (CRP). **(A)** Overall survival of 213 patients with primary MDS was stratified by PLR ≤22.14 vs. PLR >22.14 (*P* = 0.002). **(B)** Overall survival of 213 patients with primary MDS was stratified by NLR ≤3.75 vs. NLR >3.75 (*P* = 0.019). **(C)** Overall survival of 213 patients with primary MDS was stratified by CRP ≤5 mg/L vs. CRP >5 mg/L (*P* < 0.0001). **(D)** Leukemia-free survival of 213 patients with primary MDS was stratified by PLR ≤22.14 vs. PLR >22.14 (*P* > 0.05). **(E)** Leukemia-free survival of 213 patients with primary MDS was stratified by NLR ≤3.75 vs. NLR >3.75 (*P* > 0.05). **(F)** Leukemia-free survival of 213 patients with primary MDS was stratified by CRP ≤5 mg/L vs. CRP >5 mg/L (*P* = 0.041).

Through the Kaplan–Meier survival analysis and log-rank test, we observed that higher levels of PLR (>22.14), NLR (>3.75), and CRP (>5 mg/L) were significantly associated with decreased OS. As shown in **Figures 2A–C** and compared with that in the low-level counterparts, the median OS was shorter in the higher PLR (19 months vs. 60 months, *P* = 0.002), NLR (11 months vs. 27 months, *P* = 0.019), and CRP (19 months vs. 44 months, *P* < 0.0001) groups. However, the status of these indicators changed when it comes to LFS. Although a shorter LFS could be predicted by higher CRP (*P* = 0.041), its association with PLR or NLR was statistically insignificant (**Figures 2D–F**).

In univariate analysis, OS was adversely associated with older age (≥60 years, *P* < 0.0001), men (*P* = 0.018), lower HB (<10 g/dl, *P* = 0.006), higher-risk IPSS-R cytogenetic (*P* = 0.016), higher BM blast percentage (>5%, *P* < 0.0001), higher IPSS-R score (*P* < 0.0001), and higher levels of PLR (*P* = 0.003), NLR (*P* = 0.023), and CRP (*P* < 0.0001).

Factors that showed a significant difference in univariate analysis (*P* < 0.2) were further multivariately analyzed to study their influence on OS. The results showed that older age (≥60 years, *P* = 0.001), men (*P* = 0.043), lower PLT (*P* = 0.046), higher BM blast percentage (>5%, *P* < 0.0001), higher-risk IPSS-R cytogenetic (*P* = 0.026), and higher levels of PLR (*P* = 0.031) and CRP (*P* = 0.029) were adverse situations and associated with a significantly worse OS in MDS patients.

In univariate analysis, LFS was inversely associated with higher BM blast percentage (>5%, *P* < 0.0001), higher IPSS-R score (*P* = 0.002), and higher CRP (*P* = 0.044). Meanwhile, multivariate analysis was conducted to figure out the association of LFS with age, NE, BM blast percentage, IPSS-R cytogenetic risk group, and CRP. The data showed that BM blast percentage (>5%, *P* = 0.001) was an adverse factor for LFS (**Table 2**).

**Table 2:** Univariate and multivariate analyses for leukemia-free survival and overall survival in 213 patients with MDS.

Variables	Univariate analysis for LFS		Multivariate analysis for LFS		Univariate analysis for OS		Multivariate analysis for OS	
	*P*-value	HR(95% CI)	*P*-value	HR(95% CI)	*P*-value	HR(95% CI)	*P*-value	HR(95% CI)
Age ≥60 (years)	0.057	2.191 (0.976–4.919)	0.173	1.802 (0.772–4.206)	<0.0001	2.094 (1.409–3.113)	0.001	2.328 (1.446–3.747)
Gender (men)	0.329	1.496 (0.666–3.360)	–	–	0.018	1.581 (1.074–2.328)	0.043	1.605 (1.014–2.539)
HB <10 g/dl	0.296	1.539 (0.686–3.455)	–	–	0.006	1.938 (1.171–3.209)	0.055	1.737 (0.988–3.051)
NE <0.8 × 10^9^/L	0.057	2.120 (0.978–4.595)	0.797	1.118 (0.477–2.622)	0.131	1.353 (0.919–1.991)	0.898	0.969 (0.602–1.561)
PLT <100 × 10^9^/L	0.211	2.159 (0.647–7.201)	–	–	0.077	1.557 (0.929–2.610)	0.046	1.916 (1.012–3.626)
BM blast >5%	<0.0001	5.469 (2.161–13.842)	0.001	6.244 (2.176–17.918)	<0.0001	3.230 (2.184–4.777)	<0.0001	2.954 (1.833–4.760)
IPSS-R, cytogenetic risk group	0.120	1.337 (0.927–1.928)	0.371	1.193 (0.811–1.755)	0.016	1.293 (1.061–1.575)	0.026	1.269 (1.029–1.565)
IPSS-R, risk category	0.002	1.842 (1.248–2.719)	–	–	<0.0001	1.783 (1.461–2.176)	–	–
PLR >22.14	0.292	1.697 (0.635–4.540)	–	–	0.003	2.028 (1.267–3.246)	0.031	1.898 (1.059–3.399)
NLR >3.75	0.353	0.044 (0.031–31.883)	–	–	0.023	2.005 (1.099–3.658)	0.121	1.867 (0.848–4.109)
CRP >5 mg/L	0.044	2.266 (1.009–4.911)	0.278	1.597 (0.686–3.719)	<0.0001	2.019 (1.386–2.941)	0.029	1.663 (1.053–2.625)

HB, hemoglobin; NE, neutrophil; PLT, platelet; BM, bone marrow; IPSS-R, Revised International Prognostic Scoring System; PLR, platelet-to-lymphocyte ratio; NLR, neutrophil-to-lymphocyte ratio; CRP, C-reactive protein.

The significant factors in univariate analysis (P < 0.2) were used to determine the influence on OS and LFS by multivariate analysis.

## Discussion

We found that elevated pretherapy levels of PLR, NLR, and CRP, which indicate a systemic inflammatory response, were significantly associated with poor outcomes based on the available data. This study aims to focus on the predictive value of these three biomarkers. Elevated PLR levels were correlated with higher BM blast percentage, more PLT count, higher level of HB, and lower ALC. Higher NLR was associated with more NE count and lower ALC. Elevated CRP levels correlated with higher BM blast percentage, higher IPSS-R score, less PLT count, and lower level of HB. Furthermore, elevation in these three indicators was correlated with a shorter survival period in MDS, indicating their predictive value in the prognosis of MDS patients. Within the normal range, higher platelet and neutrophil counts favor better prognosis in MDS. PLR and NLR were constituted of platelet count, neutrophil count, and lymphocyte count, which can more precisely reflect disease progression than these three indicators separately. Additionally, higher CRP was associated with a shorter LFS. The Cox regression analysis revealed that the PLR and CRP levels were independent prognostic factors for MDS patients. Thus, these data suggested that PLR and CRP outweighed NLR in terms of prognostic value.

The underlying causes of MDS heterogeneity included genomic, epigenetic, bone marrow microenvironment, and autoimmune abnormalities (21). Recently, mutations such as *TP53*, *SRSF2*, *IDH2*, and *ASXL1* were also demonstrated to be valuable in predicting the prognosis of MDS (22–24). As components of the tumor microenvironment, tumor-associated inflammatory cells play an important role in tumor development (25). In recent years, the importance of patient-related factors has been recognized, particularly those involved in response to systemic inflammation that determines disease outcomes in cancer patients (26). The evidence connecting inflammation and cancer is now clearly established with the description of inflammatory cytokines that affect carcinogenesis, dedifferentiation, and primary tumor growth (27). The host inflammatory response plays an important role in the development and progression of cancer. Chronic inflammation contributes to cancer development *via* multiple mechanisms. One potential mechanism is that chronic inflammation generates an immunosuppressive microenvironment for tumor formation and progression (28). Elevation in the systemic inflammation markers is thought to reflect the activation of the innate immune/inflammation cascade in patients (29). The inflammatory bone marrow microenvironment in MDS patients may cause deterioration in clonal hematopoiesis. Chronic inflammation was identified as the key process responsible for immunosuppression *via* induction of immature myeloid-derived suppressor cells (30). Previous reports suggested that an abnormal cytokine profile was associated with the prognosis of MDS (17, 31).

The systemic inflammatory response can be reflected by peripheral blood counts and subtypes, which have the potential to predict disease prognosis. Among those biomarkers, the PLR, NLR, and CRP are derived from hematological components of the systemic inflammatory response that can reflect the inflammatory state between the tumor and host. CRP represents a sensitive marker of the inflammatory process. It follows from some studies that higher CRP levels predicted a worse prognosis in oncology (32). In a previous study, the results showed that an increased level of CRP predicts poor prognosis in low-risk MDS patients (33). Recently, both PLR and NLR have been demonstrated to be risk factors for adverse outcomes in several malignancies (34, 35). PLR and NLR are calculated by division of platelet counts and neutrophil counts, and lymphocyte counts, thus are more accurate than these three separated indicators in reflecting disease progression. Under normal circumstances, PLR and NLR maintain a relative dynamic balance. Based on the results of our study, higher levels of PLR, NLR, and CRP were associated with worse prognosis. However, high PLR and CRP levels were independent prognostic factors. Furthermore, a higher CRP level correlated with a higher incidence of leukemia transformation. In addition, the study about PLR and NLR in diseases has been on the rise in recent years, and there are no precise cutoff values for PLR and NLR. Due to the limitation in sample quantity, large-scale data are needed urgently for verification.

The present study had the following limitations. Firstly, other inflammatory indicators, such as cytokines and erythrocyte sedimentation rate, had not been measured in our study. Secondly, various gene mutations were linked to MDS prognosis; however, in our cohort, the number of patients who had received gene mutation analysis was modest, with just 60 cases being tested. The mutations were not taken into account in the Cox regression analysis. Thirdly, this study was a retrospective study, the comorbidities at diagnosis were limited to the information obtained from medical records. Lastly, this was an exploratory study with a small sample size. The result would be more convincing if the sample size had been increased. Therefore, larger prospective studies are required to confirm these preliminary results, and an investigation of the relationship between peripheral inflammatory markers and prognosis of the tumor could further expand our understanding of MDS. It is also worth mentioning, Bektaş et al. (36) pointed out that both WPSS and IPSS-R were superior predictors for OS in MDS patients, with WPSS being more effective in predicting LFS. At present, the IPSS-R system is widely used. The indicators in our study were more valuable for predicting OS, and IPSS-R differentiated chromosome karyotypes more finely than WPSS.

Despite these limitations, the results of this study suggested that PLR and CRP were independent prognostic indicators for OS in MDS. In the clinics, MDS patients with increased inflammatory levels should be properly managed, as they have a risk of poor, although other possible explanations may also contribute to their variations. When risk-stratifying patients with MDS, inflammatory cytokines are potentially valuable adjuncts to IPSS-R. Nevertheless, our findings should be confirmed in large-scale studies that include more demographic, clinical, and laboratory parameters. Imminently, assessment and treatment are critical for favorable outcomes in MDS patients. Incorporation of PLR and CRP to the traditional IPSS-R might increase its predictive performance. The clinical severity of the disease should be considered when utilizing these parameters to predict patient outcomes.

Considering the cost-effectiveness and feasibility, these markers will be applicable for a large number of patients with MDS in the near future and further be integrated into customized treatment decision-making for MDS patients.

## Data Availability Statement

The original contributions presented in the study are included in the article/supplementary material. Further inquiries can be directed to the corresponding authors.

## Ethics Statement

All patients were given a written informed consent. The project was approved by the Ethics Committee of Ningbo First Hospital (2021RS086) and was following the Declaration of Helsinki. All co-authors were included in this authorization request to have access to the data.

## Author Contributions

CS and SG collected and analyzed the data and wrote the article. QM and GO designed the research and reviewed the article. AW, TN, TL, SY, and XZ collected the data. All authors read and approved the final article.

## Funding

This research was supported by the Natural Science Foundation of Zhejiang Province (LY20H080001, LQ21H160011), the Medical and Health Plan of Zhejiang (2021KY990, 2021KY997), and the Natural Science Foundation of Ningbo (2018A610391, 2019A610306).

## Conflict of Interest

The authors declare that the research was conducted in the absence of any commercial or financial relationships that could be construed as a potential conflict of interest.

## Publisher’s Note

All claims expressed in this article are solely those of the authors and do not necessarily represent those of their affiliated organizations, or those of the publisher, the editors and the reviewers. Any product that may be evaluated in this article, or claim that may be made by its manufacturer, is not guaranteed or endorsed by the publisher.

## Abbreviations

MDS, myelodysplastic syndrome; OS, overall survival; LFS, leukemia-free survival; IPSS-R, Revised International Prognostic Scoring System; AML, acute myeloid leukemia; IPSS, International Prognostic Scoring System; WHO, World Health Organization; BM, bone marrow; HSCT, hemopoietic stem cell transplantation; ISCN2016, 2016 International System for Human Cytogenetic Nomenclature; NE, neutrophil; HB, haemoglobin; PLT, platelet; ALC, absolute lymphocyte count; AMC, absolute monocyte count; PLR, platelet-to-lymphocyte ratio; NLR, neutrophil-to-lymphocyte ratio; CRP, C-reactive protein; MDS-SLD, MDS with single-lineage dysplasia; MDS-MLD, MDS with multilineage dysplasia; MDS-RS-SLD, MDS with ring sideroblasts and single-lineage dysplasia; MDS-RS-MLD, MDS with ring sideroblasts and multilineage dysplasia; MDS-EB, MDS with excess blasts; MDS-U, unclassifiable MDS.

## References

[1] Montalban-Bravo G Garcia-Manero G. Myelodysplastic Syndromes: 2018 Update on Diagnosis, Risk-Stratification and Management. Am J Hematol (2018) 93(1):129–47. doi: 10.1002/ajh.24930 29214694

[2] Greenberg P Cox C LeBeau MM Fenaux P Morel P Sanz G. International Scoring System for Evaluating Prognosis in Myelodysplastic Syndromes. Blood (1997) 89(6):2079–88. doi: 10.1182/blood.V89.6.2079 9058730

[3] Malcovati L Germing U Kuendgen A Matteo G Cristiana P Rosangela I. Time-Dependent Prognostic Scoring System for Predicting Survival and Leukemic Evolution in Myelodysplastic Syndromes. J Clin Oncol (2007) 25(23):3503–10. doi: 10.1200/JCO.2006.08.5696 17687155

[4] Kantarjian H O'Brien S Ravandi F Cortes J Shan J Bennett M. Proposal for a New Risk Model in Myelodysplastic Syndrome That Accounts for Events Not Considered in the Original International Prognostic Scoring System. Cancer (2008) 113(6):1351–61. doi: 10.1002/cncr.23697 PMC4188533 18618511

[5] Greenberg PL Tuechler H Schanz J Sanz G Garcia-Manero G Sole F. Revised International Prognostic Scoring System for Myelodysplastic Syndromes. Blood (2012) 120(12):2454–65. doi: 10.1182/blood-2012-03-420489 PMC4425443 22740453

[6] Jacobs NL Holtan SG Porrata LF Markovic SN Tefferi A Steensma DP. Host Immunity Affects Survival in Myelodysplastic Syndromes: Independent Prognostic Value of the Absolute Lymphocyte Count. Am J Hematol (2010) 85(3):160–3. doi: 10.1002/ajh.21618 20131304

[7] Saeed L Patnaik MM Begna KH AI-Kali A Litzow MR Hanson CA. Prognostic Relevance of Lymphocytopenia, Monocytopenia and Lymphocyte-to-Monocyte Ratio in Primary Myelodysplastic Syndromes: A Single Center Experience in 889 Patients. Blood Cancer J (2017) 7(3):e550. doi: 10.1038/bcj.2017.30 28362440 PMC5380913

[8] Wu A Gao P Wu N Shi C Huang Z Rong C. Elevated Mature Monocytes in Bone Marrow Accompanied With a Higher IPSS-R Score Predicts a Poor Prognosis in Myelodysplastic Syndromes. BMC Cancer (2021) 21(1):546. doi: 10.1186/s12885-021-08303-8 33985456 PMC8117396

[9] Janssen JW Buschle M Layton M Derxler HG Lyons J van den Berghe H. Clonal Analysis of Myelodysplastic Syndromes: Evidence of Multipotent Stem Cell Origin. Blood (1989) 73(1):248– 254. doi: 10.1182/blood.V73.1.248.248 2562924

[10] Ganan-Gomez I Wei Y Starczynowski DT Colla S Yang H Cabrero-Calvo M. Deregulation of Innate Immune and Inflammatory Signaling in Myelodysplastic Syndromes. Leukemia (2015) 29(7):1458–69. doi: 10.1038/leu.2015.69 PMC4857136 25761935

[11] Papaemmanuil E Gerstung M Malcovati L Tauro S Gundem G Loo P. Clinical and Biological Implications of Driver Mutations in Myelodysplastic Syndromes. Blood (2013) 122(22):3616–27. doi: 10.1182/blood-2013-08-518886 PMC3837510 24030381

[12] Fain O Braun T Stirnemann J Fenaux P. Systemic and Autoimmune Manifestations in Myelodysplastic Syndromes. Rev Med Interne (2011) 32(9):552–9. doi: 10.1016/j.revmed.2010.08.005 20850913

[13] Kristinsson SY Bjorkholm M Hultcrantz M Derolf AR Landgren O Goldin LR. Chronic Immune Stimulation Might Act as a Trigger for the Development of Acute Myeloid Leukemia or Myelodysplastie Syndromes. J Clin Oncol (2011) 29(21):2897–903. doi: 10.1200/JCO.2011.34.8540 PMC3138717 21690473

[14] Deng Q He B Liu X Yue J Ying H Pan Y. Prognostic Value of Pre-Operative Inflammatory Response Biomarkers in Gastric Cancer Patients and the Construction of a Predictive Model. J Transl Med (2015) 18:13:66. doi: 10.1186/s12967-015-0409-0 PMC4343078 25885254

[15] Song W Tian C Wang K Zhang R Zou S. Preoperative Platelet Lymphocyte Ratio as Independent Predictors of Prognosis in Pancrcatic Cancer:A Systematic Review and Meta-Analysis. Plos One (2017) 12(6):0178762. doi: 10.1371/journal.pone.0178762. PMC5456351 28575033

[16] Steffens S Kohler A Rudolph R Eggers H Seidel C Janssen M. Validation of CRP as Prognostic Marker for Renal Cell Carcinoma in a Large Series of Patients. BMC Cancer (2012)12(1):1–7. doi: 10.1186/1471-2407-12-399 22958305 PMC3502607

[17] Shi X Zheng Y Xu L Cao C Dong B Chen X. The Inflammatory Cytokine Profile of Myelodysplastic Syndromes: A Meta-Analysis. Med (Baltimore) (2019) 98(22):e15844. doi: 10.1097/MD.0000000000015844 PMC6708708 31145332

[18] Arber DA Orazi A Hasserjian R Thiele J Borowitz M Beau M. The 2016 Revision to the World Health Organization Classification of Myeloid Neoplasms and Acute Leukemia. Blood (2016) 127(20):2391–405. doi: 10.1182/blood-2016-03-643544 27069254

[19] McGowan-Jordan J Simons A Schmid M. ISCN 2016: An International System for Human Cytogenomic Nomenclature (2016). Basel: S KARGER AG, (2016).

[20] Camp RL Dolled-Filhart M Rimm DL. X-Tile: A New Bio-Informatics Tool for Biomarker Assessment and Outcome-Based Cut-Point Optimization. Clin Cancer Res (2004) 10(21):7252–9. doi: 10.1158/1078-0432.CCR-04-0713 15534099

[21] Xu F Li X. The Role of Histone Methyltransferase EZH2 in Myelodysplastic Syndromes. Expert Rev Hematol (2012) 5(2):177–85. doi: 10.1586/ehm.12.5 22475286

[22] Tefferi A Lasho TL Patnaik MM Saeed L Mudireddy M Idossa D. Targeted Next-Generation Sequencing in Myelodysplastic Syndromes and Prognostic Interaction Between Mutations and IPSS-R. Am J Hematol (2017) 92(12):1311–7. doi: 10.1002/ajh.24901 28875545

[23] Arbab Jafari P Ayatollahi H Sadeghi R Sheikhi M Asghari A. Prognostic Significance of SRSF2 Mutations in Myelodysplastic Syndromes and Chronic Myelomonocytic Leukemia: A Meta-Analysis. Hematol (Amsterdam Netherl) (2018) 23(10):778–84. doi: 10.1080/10245332 29757120

[24] Lin P Luo Y Zhu S Maggio D Yang H Hu C. Isocitrate Dehydrogenase 2 Mutations Correlate With Leukemic Transformation and are Predicted by 2-Hydroxyglutarate in Myelodysplastic Syndromes. J Cancer Res Clin Oncol (2018) 144(6):1037–47. doi: 10.1007/s00432-018-2627-3 PMC11813452 29549529

[25] Carbone A Tripodo C Carlo-Stella C Santoro A Gloghini A. The Role of Inflammation in Lymphoma. Adv Exp Med Biol (2014) 816:315–33. doi: 10.1007/978-3-0348-0837-8_12 24818728

[26] Hanahan D Weinberg RA. Hallmarks of Cancer: The Next Generation. Cell (2011) 144(5):646±74. doi: 10.1016/j.cell.2011.02.013 21376230

[27] Demaria S Pikarsky E Coussens LM Chen YC EI-Omar EM Trinchieri G. Cancer and Inflammation: Promise for Biologic Therapy. J Immunother (2010) 33(4):335–51. doi: 10.1097/CJI.0b013e3181d32e74 PMC2941912 20386472

[28] Wang D DuBois RN. Immunosuppression Associated With Chronic Inflammation in the Tumor Microenvironment. Carcinogenesis (2015) 36:1085–1093.9. doi: 10.1093/carcin/bgv123 26354776 PMC5006153

[29] Roxburgh CS McMillan DC. Cancer and Systemic Inflammation: Treat the Tumour and Treat the Host. Br J Cancer (2014) 110(6):1409±12. doi: 10.1038/bjc.2014.90 24548867 PMC3960633

[30] Meirow Y Kanterman J Baniyash M. Paving the Road to Tumor Development and Spreading: Myeloid-Derived Suppressor Cells are Ruling the Fate. Front Immunol (2015) 6:523. doi: 10.3389/fimmu.2015.00523 26528286 PMC4601280

[31] Pardanani A Finke C Lasho TL AI-Kali A Begna KH Hanson CA. IPSS-Independent Prognostic Value of Plasma CXCL10, IL-17 and IL-6 Levels in Myelodysplastic Syndromes. Leukemia (2012) 26(4):693–9. doi: 10.1038/leu.2011.251 PMC3364441 21912394

[32] Deme D Telekes A. Prognostic Importance of Plasma C-Reactive Protein (CRP) in Oncology. Orv Hetil (2017) 158(7):243–56. doi: 10.1556/650.2017.30646 28462626

[33] Baba Y Saito B Shimada S Sasaki Y Fujiwara S Arai N. Increased Serum C-Reactive Protein Is an Adverse Prognostic Factor in Low-Risk Myelodysplastic Syndromes. Int J Hematol (2021) 114(4):441–8. doi: 10.1007/s12185-021-03187-7 34227058

[34] Walsh SR Cook EJ Goulder F Justin T A Keeling N J. Neutrophil-Lymphocyte Ratio as a Prognostic Factor in Colorectal Cancer. J Surg Oncol (2005) 91(3):181–4. doi: 10.1002/jso.20329 16118772

[35] Smith RA Bosonnet L Raraty M Sutton R Neoptolemos JP Campbell F. Preoperative Platelet-Lymphocyte Ratio is an Independent Significant Prognostic Marker in Resected Pancreatic Ductal Adenocarcinoma. Am J Surg (2009) 197(4):466–72. doi: 10.1016/j.amjsurg.2007.12.057 18639229

[36] Bektaş Ö Üner A Eliaçık E Uz B Işık A Etgül S. Comparison of Myelodysplastic Syndrome Prognostic Scoring Systems. Am J Surg (2016) 33(2):119–26. doi: 10.4274/tjh.2014.0455 PMC5100722 26376664

